# Correction to “Mitochondrial Dynamics as a Pathobiological Mediator of Clonal Myeloid Disorders”

**DOI:** 10.1111/cas.70295

**Published:** 2025-12-06

Hayashi Y, Harada H. Mitochondrial Dynamics as a Pathobiological Mediator of Clonal Myeloid Disorders. *Cancer Sci*. 2023; 114: 2722‐2728. doi: 10.1111/cas.15810.

In the above article, figure 2 is incorrect. The correct image is shown below:

**Figure:**
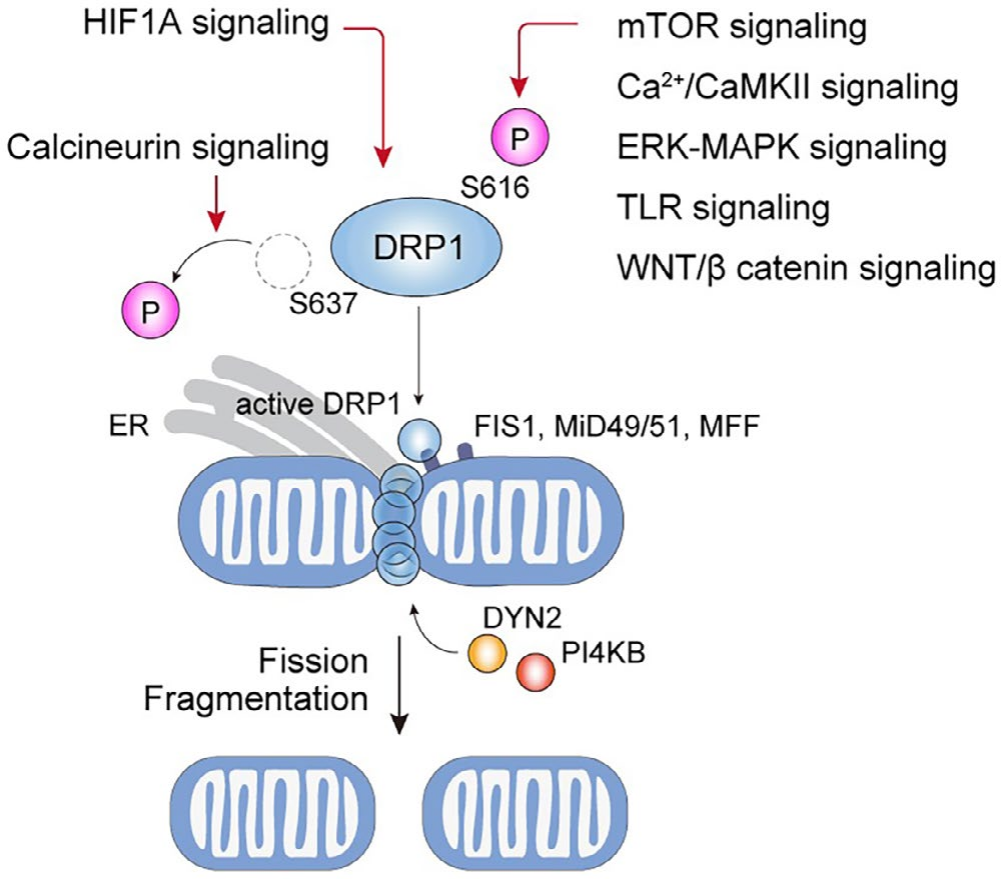
FIGURE 2. Signaling pathways regulating mitochondrial fission.

We apologize for this error.

